# The complete chloroplast genome of *Flemingia macrophylla* (Willd.) Prain (Fabaceae) from Guangxi, China

**DOI:** 10.1080/23802359.2021.1997124

**Published:** 2021-11-10

**Authors:** Xin-mei Qin, Hong Li, Nan Cui, Shui-Yuan Jiang, Yan-Ni Liang, Man-Lian Wang, Xi-Yang Huang

**Affiliations:** a Guangxi Key Laboratory of Functional Phytochemicals Research and Utilization, Guangxi Institute of Botany, Guangxi Zhuang Autonomous Region and Chinese Academy of Sciences, Guilin, China; b Wuzhou University, College of Chemical Engineering and Resource Reuse, Wuzhou, China

**Keywords:** Chloroplast genome, *Flemingia macrophylla*, phylogenetic analysis

## Abstract

*Flemingia macrophylla* (Willd.) Prain is an ethnomedicinal plant with high nutritional and medicinal values. In this study, we report the complete chloroplast genome of *F. macrophylla*. The chloroplast genome has a typical quadripartite structure with a genome size of 152,988 bp, including a large single-copy (LSC) of 83,634 bp, a small single-copy (SSC) of 17,774 bp and two inverted repeats (IRs) of 25,790 bp. The genome contains 129 genes, including 84 protein-coding, 37 tRNA and 8 rRNA genes. The overall GC content is 35.1%. Phylogenetic analysis showed that *F. macrophylla* grouped with a clade containing the genera of *Fagelia*, *Dolichos*, *Eriosema*, *Dunbaria* and *Cajanus* in Fabaceae. This study provides essential data and insight for understanding the phylogenetic placement of *Flemingia*.

The root of *Flemingia macrophylla* (Willd.) Prain 1897 classified in the Fabaceae (Xu et al. 2010) are used in traditional medicine as documented in the Pharmacopeia of the People’s Republic of China (Volume I, 2015 Edition) (Chinese Pharmacopoeia Commission 2015). The plant has also been widely used as ethnomedicine and for a diet to treat rheumatic bone pain, lumbar muscle strain, Kala-azar, fever, etc (Rana et al. 2011). So far, there has been no genome-scale (phylogenetic) study of *Flemingia.* As plastid genomes have been widely applied for phylogenetic reconstruction, species identification, population genetics and selection test (Mehmood, Abdullah Ubaid Z, Bao, et al. 2020, Mehmood, Abdullah Ubaid Z, Shahzadi, et al. 2020). In this study, we report the complete chloroplast genome sequence of *F. macrophylla* and its phylogenetic relationship to closely related genera in Papilionoideae.

Total genomic DNA was extracted from the silica-dried leaves of *F. macrophylla* using the CTAB method (Doyle 1987), which were collected from a transplanted individual in Guilin Botanical Garden (25.0704 N, 110.2991 E). The voucher specimen was deposited at the Herbarium of Guangxi Institute of Botany (http://www.gxib.cn/spIBK/, Contact person name: Chun-Rui Lin, Email: chunruilin@tom.com) under the voucher number IBK00432997. The high throughput genomic sequencing with paired ends (PE150) was performed on a NovaSeq 6000 (in Novogene corp., Tianjin, China). Approximately 3 Gb of clean data was obtained after quality filtering using fastp (Chen et al. 2018). The chloroplast genome of *F. macrophylla* was assembled with default settings using SPAdes 3.11.0 (Bankevich et al. 2012) and annotated using PGA (Qu et al. 2019). Analysis of the boundaries between IRs and single-copy regions was performed by using the online program IRSCOPE (Amiryousefi et al. 2018). The average coverage depth was calculated by mapping all the raw reads without trimming to the de novo assembled chloroplast genome in BWA-MEM (Li 2013) and SAMtools (Danecek et al. 2021). The complete chloroplast genome sequence of *F. macrophylla* was submitted to GenBank (accession number: MZ274347).

The calculated average coverage depth of *F. macrophylla* is 507 X. The chloroplast genome has a typical quadripartite structure, with a total length of 152,988 bp and an overall GC content of 35.1%, which contains one LSC region (83,634 bp), one SSC region (17,774 bp) and two IR regions (25,790 bp, respectively). It contains 129 genes, including 84 protein-coding, 8 rRNA and 37 tRNA genes. Among them, 15 genes contain one intron, and two genes contain two introns. Analysis of the boundaries between the IRs and single-copy regions of *F. macrophylla* indicated that the *rps19* gene spans the LSC/IRb boundary with a length of 230 bp in the LSC and 49 bp in the IRb; the ycf1 gene spans the SSC/IRa boundary with a length of 4848 bp in the SSC and 495 bp in the IRa; a pseudogene (ψ*ycf1*) lies at the IRb/SSC boundary, and the *trnH* gene is 30 bp away from the LSC/IRa junction. The structure of this chloroplast genome is generally in line with others of Papilionoideae reported, with a few minor differences, such as for the location of the *rps19* gene, which spans the LSC and IRb region in *F. macrophylla* but is wholly contained in the LSC region in most other species of Papilionoideae that have been reported (Zha et al. 2020).

To reveal the phylogenetic position of *F. macrophylla*, a maximum likelihood tree was reconstructed using RAxML (Stamatakis 2014) with the GTR + GAMMA substitution model based on the concatenated data of 77 protein-coding genes from the chloroplast genome sequences of 18 species. *Kennedia prostrata* was designated as the outgroup, and the tree was evaluated based on 1,000 bootstrap replicates. The phylogenetic analysis fully resolved the phylogeny and indicated that *F. macrophylla* grouped with a clade containing *Fagelia bituminosa*, *Dolichos falciformis*, *Eriosema crinitum*, *Dunbaria nivea*, *Cajanus crassus*, *Cajanus cajan* and *Cajanus scarabaeoides* (Figure 1). This is consistent with other studies based on nuclear genes from transcriptomes and/or genomes of 333 genera of Fabaceae, in which *F. macrophylla* was also suggested to be closely related to a clade consisting of *Dolichos*, *Dunbaria*, *Cajanus* and *Rhynchosia* (Zhao et al. 2021).

**Figure 1.**
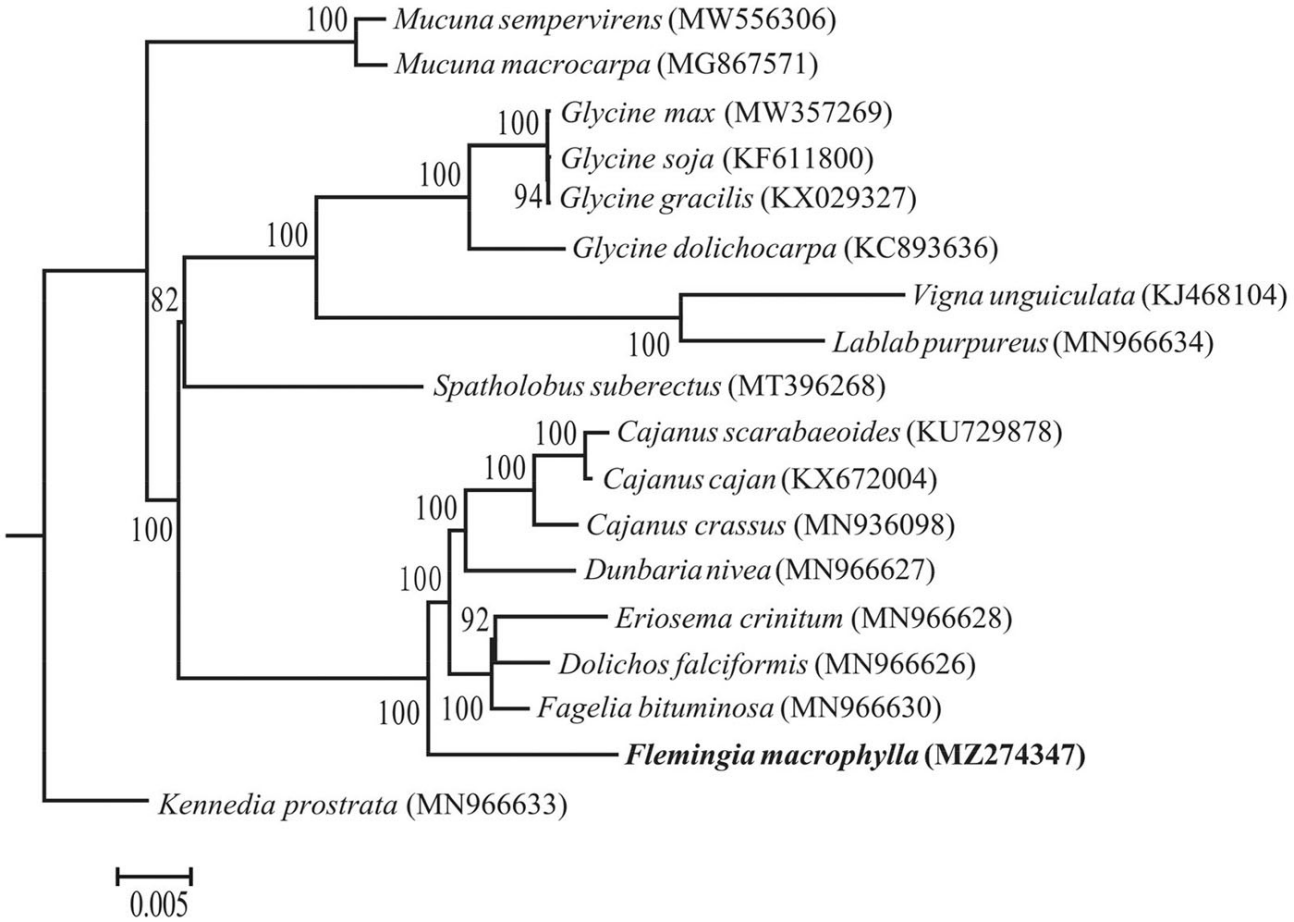
ML phylogenetic tree of the 18 species reconstructed based on the concatenated data of 77 protein-coding genes. Bootstrap support values (1000 replicates) are shown at the nodes.

## Data Availability

The genome sequence data that supports the findings of this study are openly available in GenBank at (https://www.ncbi.nlm.nih.gov/) under the accession no. MZ274347. The associated BioProject, SRA, and Bio-Sample numbers are PRJNA732804, SRR14663578, and SAMN19341677, respectively.
